# BMI Is Associated With Increased Plasma and Urine Appearance of Glucosinolate Metabolites After Consumption of Cooked Broccoli

**DOI:** 10.3389/fnut.2020.575092

**Published:** 2020-09-24

**Authors:** Craig S. Charron, Bryan T. Vinyard, Elizabeth H. Jeffery, Sharon A. Ross, Harold E. Seifried, Janet A. Novotny

**Affiliations:** ^1^US Department of Agriculture, Agricultural Research Service, Beltsville Human Nutrition Research Center, Beltsville, MD, United States; ^2^Statistics Group, US Department of Agriculture, Agricultural Research Service, Beltsville, MD, United States; ^3^Department of Food Science and Human Nutrition, University of Illinois, Urbana, IL, United States; ^4^Division of Cancer Protection, National Institutes of Health, National Cancer Institute, Rockville, MD, United States

**Keywords:** broccoli, glucosinolates, isothiocyanates, BMI, bioavailability

## Abstract

**Introduction:** Preclinical studies suggest that brassica vegetable diets decrease cancer risk, but epidemiological studies show varied effects, resulting in uncertainty about any health impact of brassicas. Factors controlling absorption of glucosinolate metabolites may relate to inconsistent results. We reported previously that subjects with BMI > 26 kg/m^2^ (HiBMI), given cooked broccoli plus raw daikon radish (as a source of plant myrosinase) daily for 17 days, had lower glucosinolate metabolite absorption than subjects given a single broccoli meal. This difference was not seen in subjects with BMI < 26 kg/m^2^ (LoBMI). Our objective in this current study was to determine whether a similar response occurred when cooked broccoli was consumed without a source of plant myrosinase.

**Methods:** In a randomized crossover study (*n* = 18), subjects consumed no broccoli for 16 days or the same diet with 200 g of cooked broccoli daily for 15 days and 100 g of broccoli on day 16. On day 17, all subjects consumed 200 g of cooked broccoli. Plasma and urine were collected for 24 h and analyzed for glucosinolate metabolites by LC-MS.

**Results:** There was no effect of diet alone or interaction of diet with BMI. However, absorption doubled in HiBMI subjects (AUC 219%, plasma mass of metabolites 202% compared to values for LoBMI subjects) and time to peak plasma metabolite values and 24-h urinary metabolites also increased, to 127 and 177% of LoBMI values, respectively.

**Conclusion:** BMI impacts absorption and metabolism of glucosinolates from cooked broccoli, and this association must be further elucidated for more efficacious dietary recommendations.

**Clinical Trial Registration:** This trial was registered at clinicaltrials.gov (NCT03013465).

## Introduction

Multiple epidemiological studies have linked consumption of brassica vegetables with reduced risk for cancers including bladder, breast, colorectal, prostate, lung, and stomach cancers (1–6). Yet other such studies have found no effect of brassica intake on cancer risk (7–10). Preclinical studies have been more consistent in finding that components of brassica vegetables have anti-cancer activity (11–15). This activity has been linked to secondary metabolites called glucosinolates. Hydrolysis of glucosinolates to bioactive isothiocyanates (ITCs), indoles, and other products is supported by myrosinase, which is endogenous to brassica plants, and by microbiota in mammalian digestive tracts.

There are numerous mechanisms by which ITCs have been shown to modulate cancer risk including inhibition of cell proliferation, induction of apoptosis, cell cycle arrest, and inhibition of histone deacetylases (16). In addition, Phase II detoxification enzymes, including the glutathione *S*-transferases (GSTs) and uridine diphosphate glucuronosyl transferases are upregulated by the action of ITCs on the nuclear factor (erythroid-derived 2)-like 2 (Nrf2) pathway. Phase II metabolism acts to conjugate xenobiotic and metabolic intermediates with sulfates, glucuronides, glutathione, or amino acids to facilitate the excretion of the conjugated molecules in urine or bile. The influence of ITCs on these biochemical pathways supports the anti-cancer health benefits of brassica vegetables and the importance of understanding how to harness these health benefits with efficacious dietary approaches.

The inconsistency of epidemiological evidence supporting the efficacy of consumption of brassica vegetables in reducing cancer risk may lie partly in the variability of absorption and metabolism of glucosinolates between and within the populations studied. Several factors may impact the absorption and metabolism of ITCs in humans. For example, there is considerable variability in glucosinolate content and myrosinase activity of brassica vegetables due to growing conditions and cultivar differences (17, 18), thereby impacting the dose of ITCs provided by the consumption of these vegetables. The ways in which brassica vegetables are prepared and cooked also affect the availability of ITCs for absorption, as the heat of cooking denatures endogenous plant myrosinase and thereby decreases ITC production from glucosinolates (19, 20). The metabolism of absorbed ITCs may be influenced by interindividual differences in glutathione *S*-transferase μ1 (GSTM1) and glutathione *S*-transferase θ1 (GSTT1) such that null versions of these genes would result in a prolonged presence of ITCs in circulation after intake of brassica vegetables (21, 22). Gut microbiota that express myrosinase-like hydrolyzing activity also may play a role in differential absorption and metabolism of ITCs by interindividual differences in the hydrolysis of glucosinolates, as evidenced by higher *ex vivo* glucoraphanin degradation by feces of high urinary ITC excreters compared to low excreters (23). Furthermore, the composition of the gut microbiota itself can be modified by the consumption of brassica vegetables (24), suggesting the possibility that frequent consumption of a diet that regularly includes brassicas may impact gut metabolism and subsequent absorption of ITCs.

We previously reported that BMI influenced the plasma ITC and metabolite response and urinary excretion after daily consumption of cooked broccoli served with raw daikon radish (25). Further, we found that repeated intake of broccoli lowered plasma metabolite and urinary excretion levels relative to levels following a single broccoli meal, but only in individuals with BMI > 26 kg/m^2^ (HiBMI). In that study, the conversion of broccoli glucosinolates to ITCs would have been catalyzed by both endogenous radish myrosinase and gut microbial myrosinase-like activity; myrosinase in commercially frozen broccoli is denatured by blanching (20). It is not known whether the observed interaction of BMI with daily feeding is facilitated by a process that depends on conversion of glucosinolates to ITCs by plant myrosinase, which was provided by the radish in that study, or if the interaction effect occurs even without the presence of plant myrosinase. Therefore, our objective was to conduct a clinical trial to investigate how daily consumption of cooked broccoli without supplementary plant myrosinase affects glucosinolate metabolism and absorption, and metabolism of resulting ITCs, including whether BMI influences plasma and/or urine metabolite response. We focused on metabolites arising from glucoraphanin, the predominant glucosinolate in broccoli, and its reduced analog, glucoerucin.

## Materials and Methods

### Subjects

**Figure 1:**
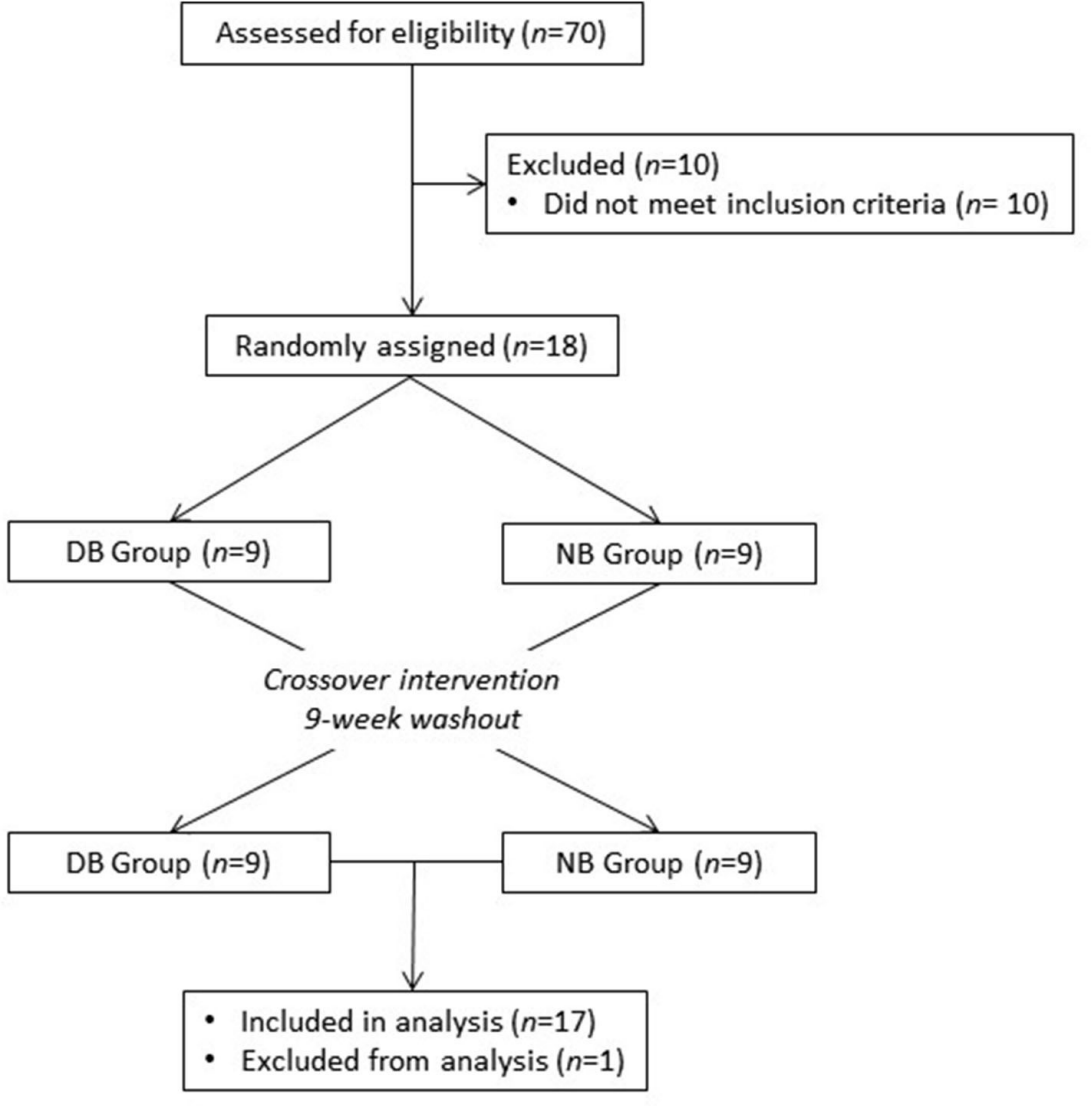
Recruitment and enrollment of subjects. One subject was excluded from analysis due to taking antibiotics during the study.

Healthy adults were recruited from the Washington, DC area to participate in this study at the Beltsville Human Nutrition Research Center (BHNRC) in Beltsville, MD, USA. Subjects were recruited from January 2017 to March 2017. Potential subjects were screened for general health by routine blood and urine screening and health history questionnaire. Applicants were excluded if they met any of the following criteria: (1) pregnant, lactating, or intending to become pregnant during the study period; (2) known allergy or intolerance to Brassica vegetables; (3) colonoscopy during 3 weeks prior to start of study; (4) use of probiotics during 3 weeks prior to start of study; (5) history of bariatric surgery or nutrient malabsorption disease (such as celiac disease) or other metabolic disorders requiring special diet recommendations; (6) use of tobacco products; (7) Crohn's disease or diverticulitis; (8) suspected or known strictures, fistulas or physiological/mechanical gastrointestinal obstruction; (9) type 2 diabetes requiring the use of diabetes pills, insulin, or non-insulin shots; (10) use of blood-thinning medications such as warfarin or anisindione; (12) self-report of alcohol or substance abuse within the past 12 months and/or current acute treatment or rehabilitation program for these problems. Potential subjects were genotyped for *GSTM1* and *GSTT1* since GST genotype can affect ITC clearance. Genotype analyses were conducted at the Bionomics Research and Technology Center of the Environmental and Occupational Health Sciences Research Institute (Piscataway, NJ) using a previously described method (26). The recruitment and enrollment data are presented in Figure 1. One subject was excluded from data analyses due to taking antibiotics during the study. Characteristics of subjects who completed the study are reported in Table 1. All subjects were *GSTT1*-present. Histograms of the distributions of subjects by age and BMI are presented in Supplementary Figures 1, 2 (Supporting Information), respectively. This study was conducted according to the guidelines laid down in the Declaration of Helsinki, and all procedures involving human subjects were approved by Chesapeake IRB (Columbia, MD, USA). Written informed consent was obtained from all subjects. This trial was registered at clinicaltrials.gov (NCT03013465).

**Table 1:** Characteristics of study participants included in analyses^a,b,c^.

**Group**	***n***	**Age (years)**	**BMI (kg/m^**2**^)**	***GSTM1* (present/null)**
**LoBMI (BMI < 26 kg/m**^**2**^**)**	8	55.3 ± 11.1	23.3 ± 2.7	3,5
Female	5	53.2 ± 13.0	23.2 ± 3.2	2,3
Male	3	58.7 ± 8.1	23.4 ± 2.1	1,2
**HiBMI (BMI > 26 kg/m**^**2**^**)**	9	53.4 ± 7.7	30.1 ± 2.6	5,4
Female	3	57.7 ± 3.1	29.9 ± 0.7	1,2
Male	6	51.3 ± 8.7	30.2 ± 3.3	4,2
**Combined**	17	54.3 ± 9.2	26.9 ± 4.3	8,9

*GSTM1, glutathione S-transferase μ 1*.

a *One subject was excluded due to taking antibiotics during the study and is not included in this table*.

b *Mean ± SD*.

c *All subjects were GSTT1-present*.

### Broccoli

Commercially blanched and frozen broccoli used in the study was procured in a single shipment prior to the start of the study. Upon arrival, broccoli was thoroughly mixed, measured into 100-g portions, and stored at −80°C until used. Glucosinolates were measured according to a previously published method (27). Two hundred grams of broccoli provided 147.6 μmol of glucoraphanin and 3.6 μmol of glucoerucin (Table 2). Fiber content was determined by the method of Prosky et al. (28).

**Table 2:** Mean daily macronutrients, fiber, glucoraphanin, and glucoerucin provided by base diet (based on 2,000 kcal) and broccoli^a^.

	**Base diet**	**Broccoli (200 g)**
	**(2,000 kcal, 8,368 kJ)**	
Protein (g)	78.3	5.8
Carbohydrate (g)	263.8	10.1
Fat (g)	70.2	0.2
Fiber (g)	18.5	5.4
Glucoraphanin (μmol)	0	147.6
Glucoerucin (μmol)	0	3.6

a *The objective of this study was to evaluate the metabolism of glucoraphanin and its reduced analog, glucoerucin. Other glucosinolates present in the broccoli (200 g) were as follows: glucobrassicin, 64.7μmol;1-methoxyglucobrassicin, 25.6 μmol; 4-hydroxyglucobrassicin, 16.2 μmol; 4-methoxyglucobrassicin, 9.1 μmol*.

### Experimental Design and Treatments

This study was a randomized, crossover design consisting of two 18-day periods separated by a 9-week washout. Power was calculated using data from our previous study (25) for the treatment × BMI interaction at hour 2, the point of maximal plasma mass of metabolites. Treatment differences of plasma mass of metabolites were calculated by comparing values at BMI = 20 kg/m^2^ to the treatment differences at BMI = 35 kg/m^2^. Based on these calculations, 16 subjects were determined to provide a statistically significant difference (α = 0.05) with power (1-β) = 95%. We enrolled 18 subjects to allow for the possibility of two drop-outs during the study, which is consistent with previous studies. Subjects consumed either a basal diet with no brassica vegetables (NB) or a daily broccoli diet (DB) for 16 days. The NB diet consisted of typical American foods providing 15.7% energy from protein, 31.6% from fat, and 52.7% from carbohydrates (Table 2). Foods were scaled in 836 kJ (200 kcal) increments to meet individual energy requirements and to maintain subject weights. One hundred grams of broccoli were consumed at breakfast and 100 g were consumed at dinner. The DB diet consisted of the basal diet plus 200 g of broccoli daily. One hundred grams portions of broccoli consumed at BHNRC were thawed at 4°C and heated in a 1,200-W microwave oven (NE-1258R; Panasonic) for 30 s, stirred, and then heated an additional 30 s, resulting in a stem core temperature of 77 ± 9°C (*n* = 5, data not shown). For broccoli consumed at home on weekends, subjects were instructed to heat 100-g portions for 30 s, stir, and repeat the cycle until the broccoli was hot but not browned or burned. On day 16 (the day preceding the pharmacokinetic challenge), subjects on the DB diet consumed 100 g of broccoli at breakfast and none at dinner. This was to minimize any carryover of metabolites to day 17, when the pharmacokinetic test was conducted. On day 17, all subjects consumed 200 g of broccoli, a 100-g roll, and 10 g of margarine for breakfast. The broccoli was heated for 60 s, stirred, and then heated an additional 60 s, resulting in a stem core temperature of 79 ± 5°C (*n* = 3, data not shown). Each subject received each dietary treatment and was randomly allocated to one of two groups using stratified randomization to balance BMI, *GSTM1* genotype, sex, and age. The allocation sequence was concealed from the principal investigator (C.S.C.) and was assigned dietary treatment order by the study coordinator. Laboratory analysts were blinded to the treatment allocation whereas subjects could not be blinded to whether they consumed daily broccoli.

Subjects were instructed to eat all foods and only foods provided by the BHNRC excepting coffee, tea, and diet soda. Breakfast and dinner were consumed at BHNRC on weekdays, and lunches and weekend meals were packed out in coolers. Coffee and tea intake were limited to 2 cups/d and diet soda was not limited. Subjects were provided a list of common brassica foods and asked to abstain from these foods and vitamin and mineral supplements beginning 3 weeks before the study and ending upon the final day of the study. No adverse effects were observed. All subjects completed the study but the data from one subject were not included in the analysis because the subject took antibiotics during the study.

### Sample Collection and Analysis

During the pharmacokinetic test initiated on day 17, blood and urine were collected for 24 h. Blood was collected immediately before the broccoli test meal, hourly from hours 1 to 14, and at hour 24. Blood was collected into EDTA-coated vacutainers and centrifuged at 2,000 g for 10 min. 1.5-ml aliquots of plasma were transferred to cryovials, snap-frozen in liquid N_2_, and stored at −80°C until analysis. Urine was collected beginning before the broccoli test meal and during the periods 0–2, 2–4, 4–6, 6–8, 8–10, 10–12, 12–14, and 14–24 h after the test meal. From 14 to 24 h, subjects collected all urine into a 3 L collection container containing 2 g of ascorbic acid, which was dispensed in 1.5 mL aliquots into cryovials with no additional acidification, and stored at −80°C. Urine collected from 0 to 14 h was aliquoted in volumes of 1.5 ml to cryovials containing 0.25 mL of 0.7% ascorbic acid and stored at −80°C.

### Analysis of Sulforaphane and Conjugates of Sulforaphane and Erucin in Plasma and Urine

The analysis of sulforaphane (SF) and conjugates of SF and erucin (ER) in plasma and urine were performed as previously described (25). Briefly, 0.5-mL aliquots of plasma were combined with 50 μL of 100-μM *N*-acetyl (*N*-butylthiocarbamoyl)-L-cysteine as an internal standard and 50 μL of trifluoracetic acid. Following centrifugation at 16,000 g for 10 min and 4°C, the supernatant was filtered with a 0.2-μm nylon spin filter at 10,000 g for 5 min at 4°C in preparation for analysis by LC-MS. 0.5-mL aliquots of thawed urine were combined with 25 μL of *N*-acetyl (*N*-butylthiocarbamoyl)-L-cysteine, centrifuged at 10,000 g for 5 min and 4°C, combined with 4.5 mL of 10-mM ammonium acetate buffer (pH 4.0), and vortex-mixed for analysis.

Analysis of plasma and urine samples was done as described previously (25) using ultra high pressure liquid chromatography (Agilent Zorbax SB-Aq column; 2.1 × 100 mm, 1.8 μm) and an Agilent 6490 triple quadrupole mass spectrometer. Selected reaction monitoring was used for the following collision induced dissociation (CID) transitions: SF (m/z 178–114), SF-glutathione (SF-GSH, m/z 485–136), SF-cysteineglycine (SF-CG, m/z 356–136), SF-cysteine (SF-C, m/z 299–136), SF-N-acetylcysteine (SF-NAC, m/z 341–178), ER-glutathione (ER-GSH, m/z 469–179), ER-cysteineglycine (ER-CG, m/z 340–179), ER-cysteine (ER-C, m/z 283–103), ER-N-acetylcysteine (ER-NAC, m/z 325–164), and *N*-acetyl (*N*-butylthiocarbamoyl)-L-cysteine (m/z 279–122). External standard curves were produced using standards synthesized and purified by previously reported methods (29, 30), and used to quantify metabolites in plasma and urine.

### Statistics

Previously developed statistical procedures were applied to the data in this study (25). Levels of metabolites were adjusted for estimated plasma volumes (31, 32) and are reported as plasma mass of metabolites (μmol). Urinary excretion rates (μmol/h) of metabolites were calculated as the ratio of metabolite concentration to the duration of urine collection. Plasma AUC was calculated using the linear trapezoidal method. The maximum plasma mass of metabolites (M_max_) and time to reach this maximum (T_max_) were determined by visual inspection of each time point curve. The elimination rate constant (k) was estimated from non-linear exponential decay curves fit to the elimination phase of plasma mass-time curves. ANOVA was performed using the GLIMMIX procedure in SAS (ver. 9.4; SAS Institute). For the 24-h urinary accumulation of the metabolites ERC, SF-C, ER-NAC, SF-NAC, and SF (expressed as proportions relative to the 24-h sum of metabolites) and for k, whose observed values were continuously distributed across a fixed-interval [i.e., (a, b) for any a < b] range, ANOVA models used a Beta distribution with a logit function to link inferences to the original scale of the observed data values. For AUC values of individual metabolites, ANOVA models used a negative binomial distribution with log link and offset log_e_(AUC) to obtain estimates as a proportion of the AUC of the sum of metabolites. For all other variables, observed values were continuously distributed like time values across a positive range (0, ∞). ANOVA models used a Gamma distribution with a log function to link inferences to the original scale of the observed data values. Plots of models' residuals were examined to verify goodness of fit. Models that analyzed plasma and urinary metabolites responses with time included the following effects in the model: subject, period, sequence, hour, diet, BMI, GSTM1 genotype, sex, diet × hour, diet × BMI, and diet × BMI × hour. All other models excluded the hour terms. Subject was modeled as a random effect and other effects were modeled as fixed effects. To graph the responses by BMI, the data were separated into two groups: one of BMI < 26.2 kg/m^2^ (LoBMI, *n* = 8) and the other of BMI > 26.2 kg/m^2^ (HiBMI, *n* = 9). After one subject was excluded from analysis due to taking antibiotics, it was not possible to divide by median the remaining 17 subjects into two groups with equal numbers of subjects. Therefore, we chose to use the median from the previous study (26.2 kg/m^2^, subsequently referred to as BMI of 26 kg/m^2^) rather than the median of this current study (26.4 kg/m^2^). All significance tests were conducted on the model's logit or log scale and reported on the original scale as least squares means with 95% CI.

## Results

**Figure 2:**
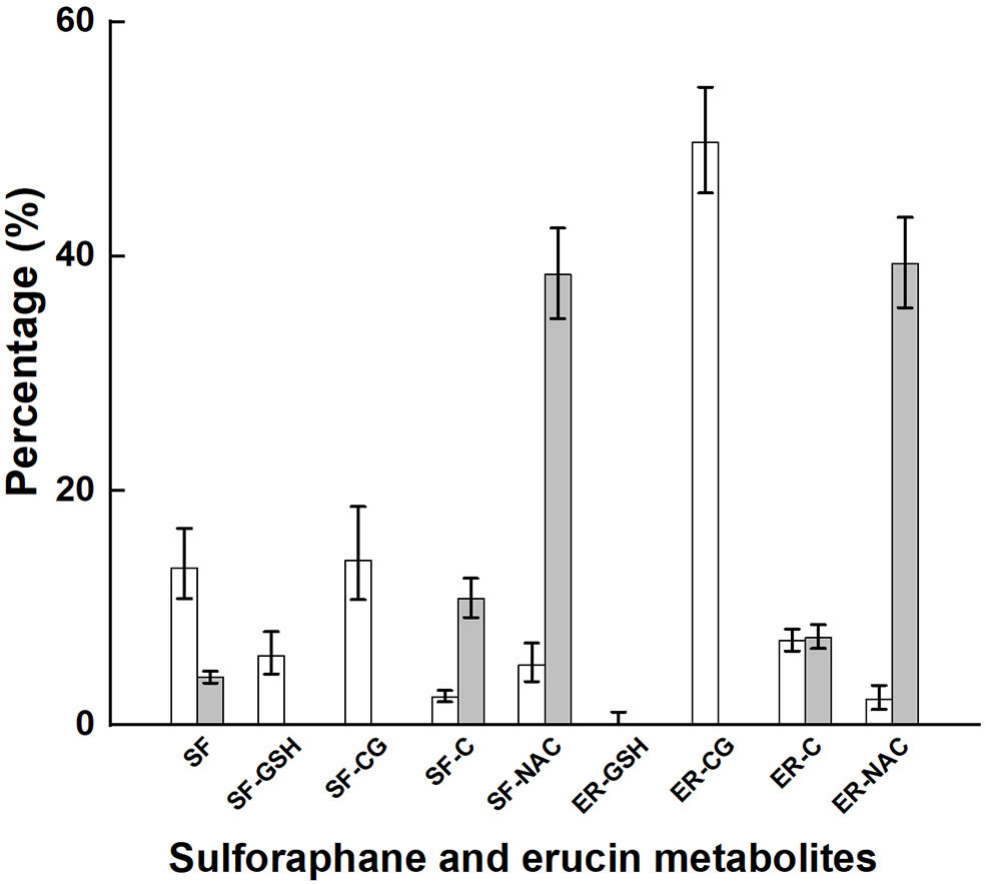
Plasma AUC and urinary accumulation over 24 h of metabolites of glucoraphanin and glucoerucin expressed as the percentage of total AUC and total urinary accumulation, respectively, of subjects who had consumed the control diet. Values are least squares means and 95% confidence intervals. SF, sulforaphane; SF-GSH, sulforaphane-glutathione; SF-CG, sulforaphane-cysteineglycine, SF-C, sulforaphane-cysteine; SF-NAC, sulforaphane-N-acetylcysteine; ER-GSH, erucin-glutathione; ER-CG, erucin-cysteineglycine; ER, erucin-cysteine; ER-NAC, erucin-N-acetylcysteine. □, percentage plasma AUC; ■, percentage urinary accumulation.

Glucoraphanin comprised 98% of the sum of glucoraphanin and glucoerucin provided by broccoli (Table 2). SF, SF-GSH, SF-CG, SF-C, SF-NAC, ER-GSH, ER-CG, ER-C, and ER-NAC were measured in plasma, and SF, SF-C, SF-NAC, ER-C, and ER-NAC were measured in urine. The proportional appearance of these metabolites in plasma and urine are presented as percentages of total plasma AUC (over 24 h) and total urinary accumulation (24 h), respectively (Figure 2, NB diet); absolute totals are given in Table 3. Results from the DB diet were similar and are presented in Supplementary Figure 3 (Supporting Information). SF and SF metabolites comprised 41 and 45% of total metabolites in plasma for the NB and DB diets, respectively, and comprised 53 and 56% of total metabolites in 24-h urine for the NB and DB diets, respectively. Specifically, the predominant metabolite in plasma was ER-CG (50% of total), followed by SF-CG (14%), SF (13%), ER-C (7%), SF-GSH (6%), SF-NAC (5%), SF-C, and ER-NAC (each 2%), and ER-GSH (<1%). In urine, the predominant metabolites were ER-NAC (39% of total) and SF-NAC (38%), followed by SF-C (11%), ER-C (7%), and SF (4%).

**Table 3:** Pharmacokinetic data following consumption of a meal of 200 g of broccoli preceded by 16 days of a control diet with no broccoli or the same control diet with daily consumption of 200 g of broccoli.

	**Plasma total metabolites**								**Urinary total**	
	**AUC (μmol·h)**		**M**_****max****_ **(μmol)**		**T**_****max****_ **(h)**		**k (h**^**−1**^**)**		**Metabolites in 24 h (μmol)**	
	**Mean**	**95% CI**	**Mean**	**95% CI**	**Mean**	**95% CI**	**Mean**	**95% CI**	**Mean**	**95% CI**
**Overall means**										
NB diet	4.7	3.5, 6.2	0.74	0.54, 1.00	5.3	4.7, 6.0	0.26^#^	0.22, 0.30	16.3	12.4, 21.5
DB diet	4.5	3.4, 5.7	0.69	0.50, 0.94	5.7	5.1, 6.4	0.21	0.18, 0.25	18.1	13.7, 23.7
**LoBMI**										
Overall	3.1**	2.1, 4.4	0.50*	0.33, 0.76	4.9*	4.2, 5.7	0.23	0.19, 0.29	12.9	8.8, 18.7
NB diet	3.4	2.2, 5.0	0.56	0.36, 0.89	4.5	3.7, 5.4	0.27	0.21, 0.33	13.2	8.6, 19.8
DB diet	2.8	1.9, 4.2	0.44	0.28, 0.70	5.3	4.4, 6.3	0.21	0.16, 0.26	12.6	8.2, 19.0
**HiBMI**										
Overall	6.8	4.8, 9.8	1.01	0.67, 1.52	6.2	5.4, 7.2	0.24	0.19, 0.28	22.8	16.0, 32.4
NB diet	6.5	4.4, 9.8	0.96	0.62, 1.49	6.3	5.3, 7.5	0.25	0.20, 0.31	20.2	13.7, 29.7
DB diet	7.1	4.8, 10.5	1.07	0.69, 1.65	6.1	5.2, 7.3	0.22	0.18, 0.28	25.7	17.5, 37.6
**Significance (*****p*****)**^**a**^										
Diet	0.644		0.551		0.311		0.039		0.360	
BMI	0.006		0.025		0.028		0.965		0.036	
Diet x BMI	0.213		0.144		0.149		0.387		0.195	

*Least squares means and 95% confidence intervals; LoBMI (BMI < 26 kg/m^2^, n = 8) and HiBMI (BMI > 26 kg/m^2^, n = 9)*.

*NB, control diet; DB, control diet with daily consumption of 200 g of broccoli; AUC, area under curve; M_max_, maximal plasma mass of metabolites; T_max_, time of maximal plasma mass of metabolites; k, elimination rate constant; Urinary total metabolites, total accumulation of urinary metabolites over 24 h*.

a *Unadjusted p-values associated with F-tests from (n = 17) crossover ANOVA model effects: diet, BMI, and diet x BMI; error term df=15*.

* *Mean value of this pharmacokinetic parameter was significantly different for LoBMI compared to HiBMI, regardless of diet (p < 0.05), determined by ANOVA model linear contrasts*.

** *Mean value of this pharmacokinetic parameter was significantly different for LoBMI compared to HiBMI, regardless of diet (p < 0.01), determined by ANOVA model linear contrasts*.

# *Mean value for NB diet was different from that for DB diet, p < 0.05*.

Of the 151.2 μmol of glucoraphanin and glucoerucin provided in the broccoli test meal, 16.3 μmol (10.8%) and 18.1 μmol (12.0%) were recovered as metabolites in 24-h urine for the NB and DB diets, respectively (*p* = 0.360) (Table 3). Although there was no diet effect, there was a significant BMI effect such that for urine from LoBMI subjects, 12.9 μmol (8.5%) of the 151.2 μmol of glucoraphanin and glucoerucin were recovered as urinary metabolites whereas urine from HiBMI subjects contained 22.8 μmol (15.1%) of the glucoraphanin and glucoerucin (*p* = 0.036). Whereas, the BMI effect was significant, the diet × BMI interaction for urinary total metabolites in 24 h was non-significant (*p* = 0.195).

**Figure 3:**
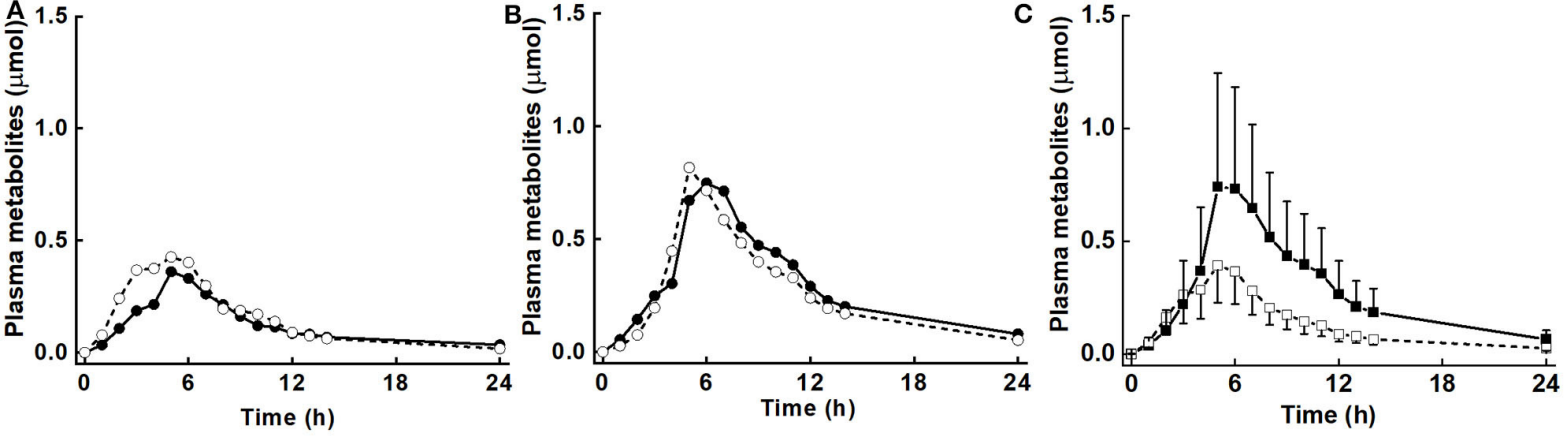
Plasma mass (μmol) of metabolites (sum of sulforaphane, sulforaphane-glutathione, sulforaphane-cysteineglycine, sulforaphane-cysteine, sulforaphane-N-acetylcysteine, erucin-glutathione, erucin-cysteineglycine, erucin-cysteine, and erucin-N-acetylcysteine) with time for control and broccoli diets. **(A)** LoBMI, BMI < 26 kg/m^2^ (*n* = 8), **(B)** HiBMI, BMI > 26 kg/m^2^ (*n* = 9), and **(C)** each BMI group with combined data from the control and broccoli diets (*n* = 17). Values are least squares means. Because the response curves within **(A,B)** are similar and error bars were overlain and obscured the response curves, the error bars are not shown. *p* = 0.147 for diet × BMI interaction as determined by ANOVA model linear contrasts. For **(C)**, upper 95% confidence limits are shown above largest mean at a given time and lower 95% confidence limits are shown below smallest mean at a given time. *p* = 0.079 for main effect of BMI. •, daily broccoli diet; °, control diet; ■, HiBMI, BMI > 26 kg/m^2^; □, LoBMI, BMI < 26 kg/m^2^.

There was no diet effect or significant interaction of diet × BMI in plasma for AUC, M_max_, or T_max_ (Table 3). However, as for urinary metabolites, there was a significant main effect of BMI for AUC, M_max_, and T_max_, and a diet effect for k. AUC was 3.1 and 6.8 μmol·h for LoBMI and HiBMI, respectively (*p* = 0.006). M_max_ was 0.5 and 1.01 μmol for LoBMI and HiBMI, respectively (*p* = 0.025). Although less divergent, T_max_ also differed by BMI. For LoBMI, T_max_ = 4.9 h and for HiBMI, T_max_ = 6.2 h (*p* = 0.028). Plots of plasma mass of metabolites vs. time (up to 24 h) are presented in Figure 3A (plasma mass of metabolites by diet for LoBMI), Figure 3B (plasma mass of metabolites by diet for HiBMI), and Figure 3C (plasma mass of metabolites by BMI with NB and DB diets combined). The effect of BMI on plasma mass of metabolites over time was non-significant (*p* = 0.079). However, the plasma mass curves are not inconsistent with Table 3 where AUC and M_max_ for LoBMI were lower than those for HiBMI. Notably, the T_max_ values observable in Figure 3C appear to be similar for LoBMI and HiBMI, in contrast to Table 3 where T_max_ is lower for LoBMI compared to T_max_ for HiBMI. This likely occurred because the T_max_ in Table 3 was determined using each individual response curve and the shapes of these curves among individuals varied. Because the T_max_ in Table 3 is the least squares mean of directly determined T_max_ values of individual subjects, it represents T_max_ independently of curve shape.

**Figure 4:**
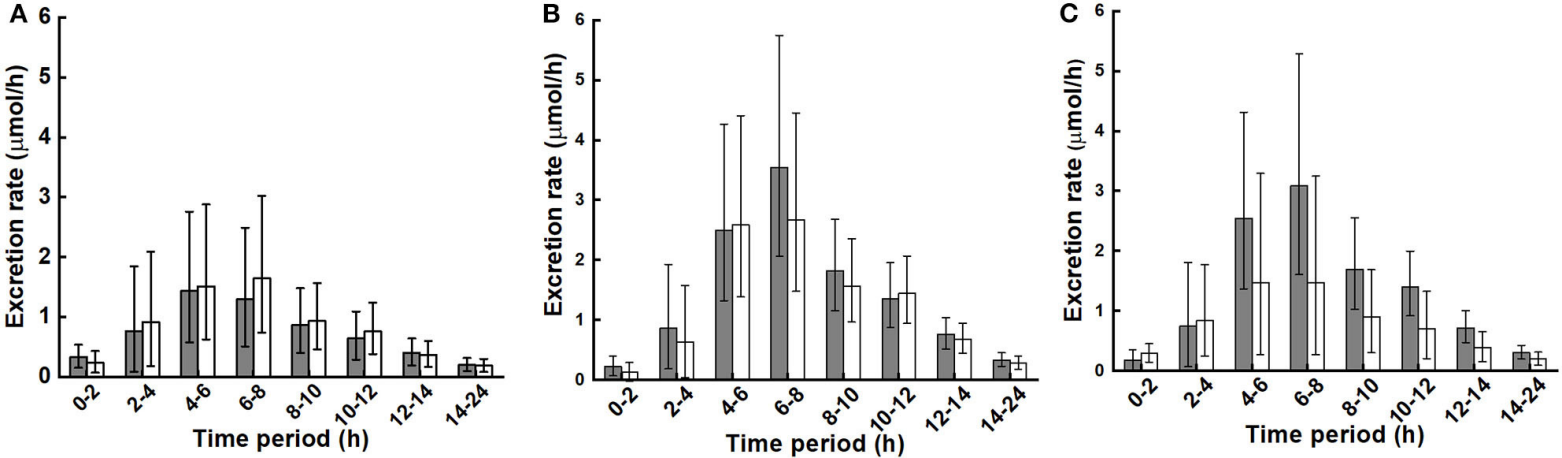
Urinary excretion rate of metabolites (sum of sulforaphane, sulforaphane-glutathione, sulforaphane-cysteineglycine, sulforaphane-cysteine, sulforaphane-N-acetylcysteine, erucin-glutathione, erucin-cysteineglycine, erucin-cysteine, and erucin-N-acetylcysteine) during collection periods for control and broccoli diets. **(A)** LoBMI, BMI < 26 kg/m^2^ (*n* = 8), **(B)** HiBMI, BMI > 26 kg/m^2^ (*n* = 9), and **(C)** each BMI group with combined data from the control and broccoli diets (*n* = 17). Values are least squares means and 95% confidence intervals. *p* = 0.072 for main effect of BMI as determined by ANOVA model linear contrasts. *p* = 0.655 for diet × BMI interaction. For **(A,B)**: ■, daily broccoli diet; □, control diet. For **(C)**: ■, HiBMI, BMI > 26 kg/m^2^; □, BMI < 26 kg/m^2^.

Urinary excretion rates during the collection time periods are shown in Figure 4. As with the plasma response over time, diet did not affect urinary excretion rates nor the interaction of diet with BMI, while the main effect of BMI was close to significance (*p* = 0.072).

## Discussion

Results indicate that in comparison to consuming a brassica-free control diet for 16 days, consuming cooked broccoli daily for 16 days prior to the broccoli test meal did not influence glucosinolate metabolism or ITC absorption and metabolism, regardless of BMI. In contrast, there was a pronounced effect of BMI on absorption and metabolism, unrelated to frequency of broccoli intake. Appearance of glucosinolate metabolites in plasma and urine was greatly increased in the HiBMI group compared to the LoBMI group. Specifically, both plasma glucosinolate metabolite AUC and M_max_ doubled in subjects with BMI > 26 kg m^−2^ (HiBMI, 30.1 ± 2.6 kg/m^2^, mean ± SD) compared to metabolite values from subjects with BMI < 26 kg/m^2^ (LoBMI, 23.3 ± 2.7 kg/m^2^, mean ± SD), even given that the number of subjects was small (*n* = 17; Table 3). Time to maximum plasma levels of metabolites, T_max_, and the 24-h accumulation of total urinary metabolites were also greater (127 and 177%, respectively) in subjects with HiBMI compared to values from subjects with LoBMI (Table 3). Furthermore, T_max_ values indicate that the peak of plasma metabolites was delayed 1.3 h in subjects with a high BMI compared to subjects with a low BMI. The impact of BMI on glucosinolate metabolism and absorption may be a confounding factor in clinical trials evaluating the health impact of broccoli feeding.

In our earlier report, we determined that AUC was 10.9 and 10.0 μmol·h for the NB and DB diets, respectively, after the broccoli + daikon radish test meal (25). In contrast, AUC in this current study was far lower, with 4.7 and 4.5 μmol·h for the NB and DB diets, respectively, despite the higher dose of glucoraphanin (148 μmol in this study compared to 98 μmol in the earlier study). The lower plasma response following a higher dose is a reflection of the importance of plant myrosinase for conversion of glucoraphanin to ITC. Expectedly, given that some hydrolysis can occur in the stomach or small intestine in the presence of plant myrosinase (in the first study) but not in the absence of plant myrosinase, T_max_ in the earlier study was 2.1 (NB diet) and 2.3 h (DB diet) whereas in the current study, T_max_ was 5.3 (NB diet) and 5.7 h (DB diet), about 3 h longer. Decreased and delayed absorption of ITCs and ITC metabolites after the consumption of cooked broccoli or boiled broccoli sprouts has been previously reported (33). In a study comparing fresh and steamed broccoli of equivalent glucosinolate levels, AUC following steamed broccoli was 42% lower than that following fresh broccoli and T_max_ was delayed from 2.1 h (fresh broccoli) to 6 h after consuming steamed broccoli (34). Others also have reported that consuming cooked broccoli results in decreased 24-h accumulation of urinary metabolites compared to fresh broccoli (35). Thus, our data are in agreement with others, that in the absence of plant myrosinase, glucosinolate metabolism and absorption is less and slower, but does occur.

We considered whether the reason for enhanced absorption and excretion in subjects with a higher BMI could be related to a prolonged gut transit time: if, for example, a longer transit time allowed for prolonged contact time with enterocytes. The later T_max_ for plasma from those with a HiBMI is consistent with a longer transit time but does not confirm it. However, contrary to this hypothesis, in studies using test meals to establish normal values in healthy individuals, gastric emptying and small-bowel transit times were found to be unrelated to BMI. In a multi-center study, scintigraphy was used to assess gastric emptying of a ^99^Tc-labeled low fat meal, which found that 50% gastric retention occurred around 1.5 h, with no effect of BMI (36). In a large retrospective study (*n* = 619) using video capsule endoscopy, small-bowel transit time was not associated with BMI (37). Nonetheless, the later T_max_ for the HiBMI group may indicate slower transit time for this group of individuals, and these results may reflect a transit time issue rather than a BMI issue. Further studies will be needed to identify the mechanism.

The plasma response curves for the LoBMI and HiBMI groups track closely from 0 to 5 h, and then diverge, with plasma levels rising at a higher rate in those with a high BMI compared to those with a lower BMI (Figure 3C). We propose that this difference in plasma levels reflects a difference in response of the colon and associated microbiota to the broccoli glucosinolates. Glucosinolates can be metabolized by gut microbial species including *Citrobacter* WYE1 and *Enterobacter cloacae* KS50 (38) and differences in fecal bacterial composition are thought to produce differences in the excretion of glucoraphanin metabolites (23). Furthermore, microbial composition is known to vary between lean and obese individuals, particularly with regard to the ratio of Firmicutes to Bacteroidetes (39, 40). To the best of our knowledge, it has not been established whether gut content of myrosinase-expressing bacteria varies with BMI. When active myrosinase is included in glucosinolate-rich meals as in our earlier study (25) and in that of Fahey et al. (41), ITCs and ITC metabolites are rapidly produced and then absorbed in the upper digestive tract. Neither study reported a main effect of BMI. Those results are not contrary to the notion that the BMI association observed in the current study was related to the lower digestive tract, and particularly to gut bacteria-mediated glucosinolate metabolism and absorption in the region of the colon.

Besides possible BMI-related differences in communities of myrosinase-expressing bacteria, there may be differences in microbial activities that direct glucosinolate metabolism toward the generation of non-bioactive nitriles at the expense of ITCs. In cell culture, *Lactobacillus plantarum* KW30 and *Lactococcus lactis* subsp. *lactis* KF147 converted glucoiberin and glucoraphanin to iberverin nitrile, erucin nitrile, and sulforaphane nitrile (42). A second pathway of nitrile formation may involve the desulfation of glucosinolates by bacteria with sulfatase activity (43) followed by the metabolism of desulfoglucosinolates to nitriles as has been observed in fermentation cultures of *E. co*li VL8, *L. agilis* R16, and *E. casseliflavus* (44). Additionally, the presence of Fe^2+^ is known to favor nitrile formation whereas Mg^2+^ increases ITC production (44). The lower levels of plasma metabolites and urinary accumulation of ITC metabolites in subjects with LoBMI may have occurred in response to higher nitrile-generating activity of gut microbiota. However, because subjects consumed a controlled diet, it is unlikely that there were differences in concentrations of Fe^2^ or Mg^2+^ ions that influenced glucosinolate hydrolysis. We did not measure nitrile content.

The strengths of this study include the randomized, crossover study design, the use of controlled feeding, the measurement of metabolites of glucoraphanin and glucoerucin, and the use of multiple collection points during the 24 h following the test meal. Also, there were limitations to the study. The unconjugated isothiocyanate erucin was not measured because it could not be reliably measured on our analytical system (25) and knowledge of erucin in plasma and/or urine may have provided more insight into glucoraphanin and glucoerucin metabolism. However, conjugated erucin metabolites were measured. Another limitation was that there was a small energy and fiber difference between the NB and DB diets due to the addition of broccoli to the DB diet. Two hundred grams of broccoli daily provided 264 kJ d^−1^ (62 kcal d^−1^). We previously calculated that this would add about 0.15 kg to subject body weight over 16 d, which for an 80-kg subject, is 0.2% of the body weight and probably is insignificant (25). The additional 5.4 g/d of fiber could have impacted the composition of the microbiome, but as there was no influence of diet on metabolite levels, this was not likely responsible for the different responses by BMI. Finally, we chose to study the predominant glucosinolate in broccoli, glucoraphanin, and glucoerucin with which it interconverts. The association of BMI with indole glucosinolate metabolites was not studied, but may be worthy of future investigation.

In conclusion, daily consumption of cooked broccoli without supplementary plant myrosinase did not affect plasma or urinary metabolite levels of glucoraphanin and glucoerucin measured following a cooked broccoli test meal consumed at the end of the adaption period. However, subjects with HiBMI had elevated levels of plasma and urinary metabolites and a delayed maximal plasma metabolite peak compared to those with LoBMI. This BMI-associated response was not related to daily broccoli consumption, as we observed in an earlier study in which supplementary plant myrosinase was provided with the broccoli. It is possible that gut transit time and/or differences in gut microbiota played a role in this population. The association of BMI with differential absorption and metabolism of ITCs warrants further investigation to ultimately provide more efficacious dietary guidance for optimal health.

## Data Availability Statement

The raw data supporting the conclusions of this article will be made available by the authors, without undue reservation.

## Ethics Statement

The studies involving human participants were reviewed and approved by Chesapeake IRB, MD, USA. The patients/participants provided their written informed consent to participate in this study.

## Author Contributions

CC, JN, EJ, BV, HS, and SR designed the research. CC and JN conducted the research. BV conducted the statistical analyses. CC drafted the article. All authors edited and approved the article.

## Conflict of Interest

The authors declare that the research was conducted in the absence of any commercial or financial relationships that could be construed as a potential conflict of interest.

## Abbreviations

DB: daily broccoli
ER: erucin
ER-C: erucin-cysteine
ER-CG: erucin-cysteineglycine
ER-GSH: erucin-glutathione
ER-NAC: erucin-*N*-acetylcysteine
GST: glutathione *S*-transferase
*GSTM1*: glutathione *S*-transferase μ 1
*GSTT1*: glutathione *S*-transferase θ 1
ITC: isothiocyanate
LSmean: least squares mean
NB: no broccoli
SF: sulforaphane
SF-C: sulforaphane-cysteine
SF-CG: sulforaphane-cysteineglycine
SF-GSH: sulforaphane-glutathione
SF-NAC: sulforaphane-*N*-acetylcysteine
ER-C: erucin-cysteine
ER-CG: erucin-cysteineglycine
ER-GSH: erucin-glutathione
ER-NAC: erucin-*N*-acetylcysteine.
