# Supplementary figures and images for: BMI Is Associated With Increased Plasma and Urine Appearance of Glucosinolate Metabolites After Consumption of Cooked Broccoli
Source: Front Nutr. 2020 Sep 24;7:575092. doi: 10.3389/fnut.2020.575092 (PMC7542245; doi:10.3389/fnut.2020.575092)

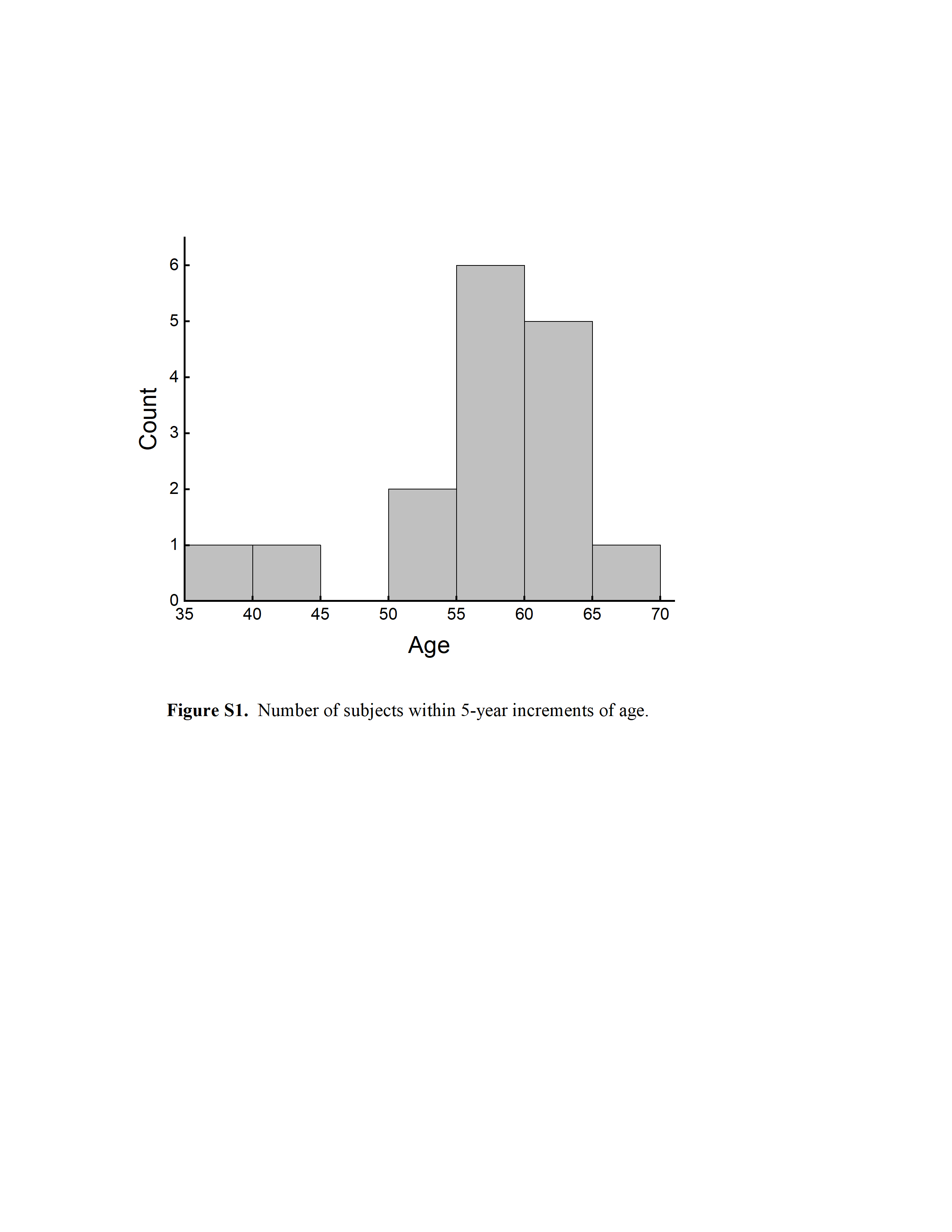

Supplement: Supplementary file 1 [file Image_1.JPEG]

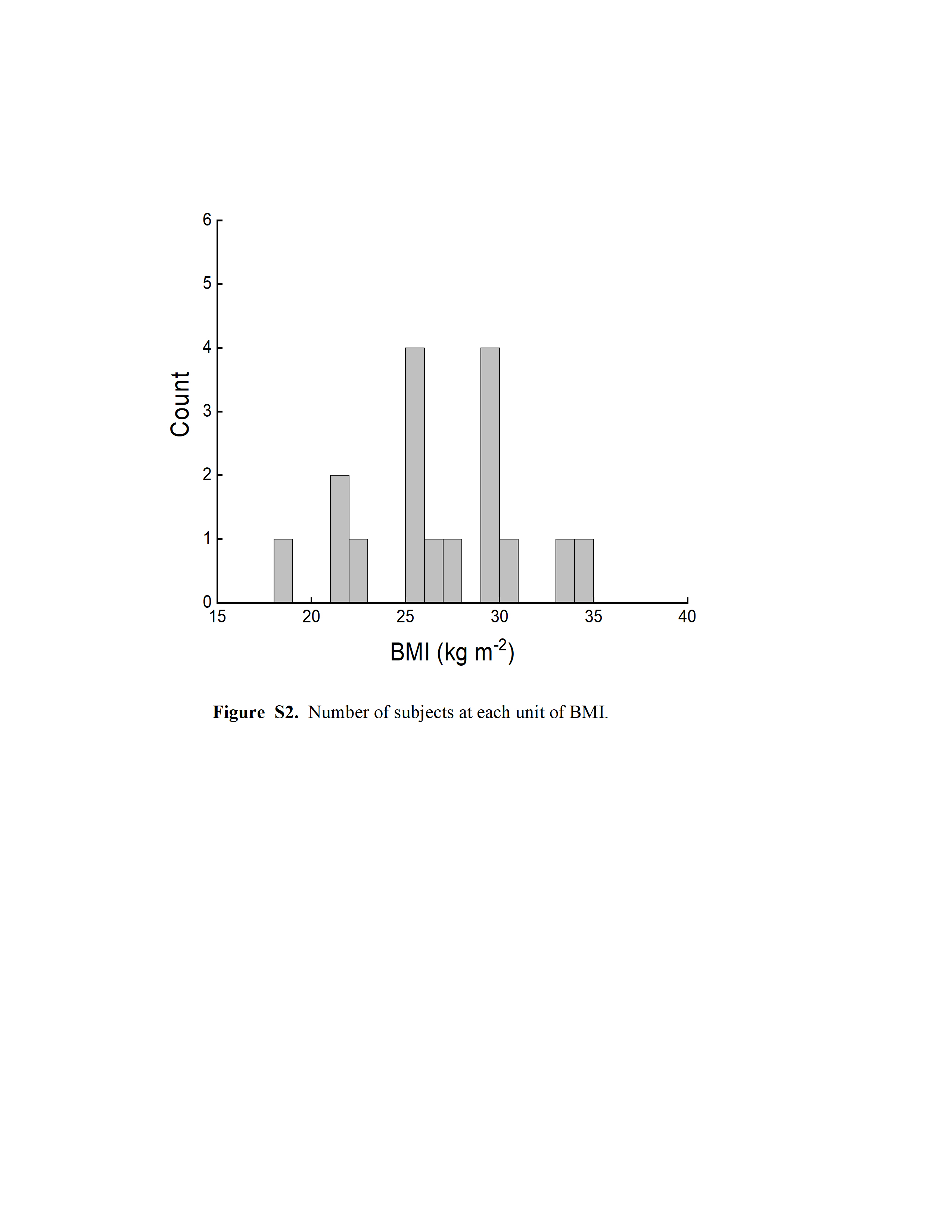

Supplement: Supplementary file 2 [file Image_2.JPEG]

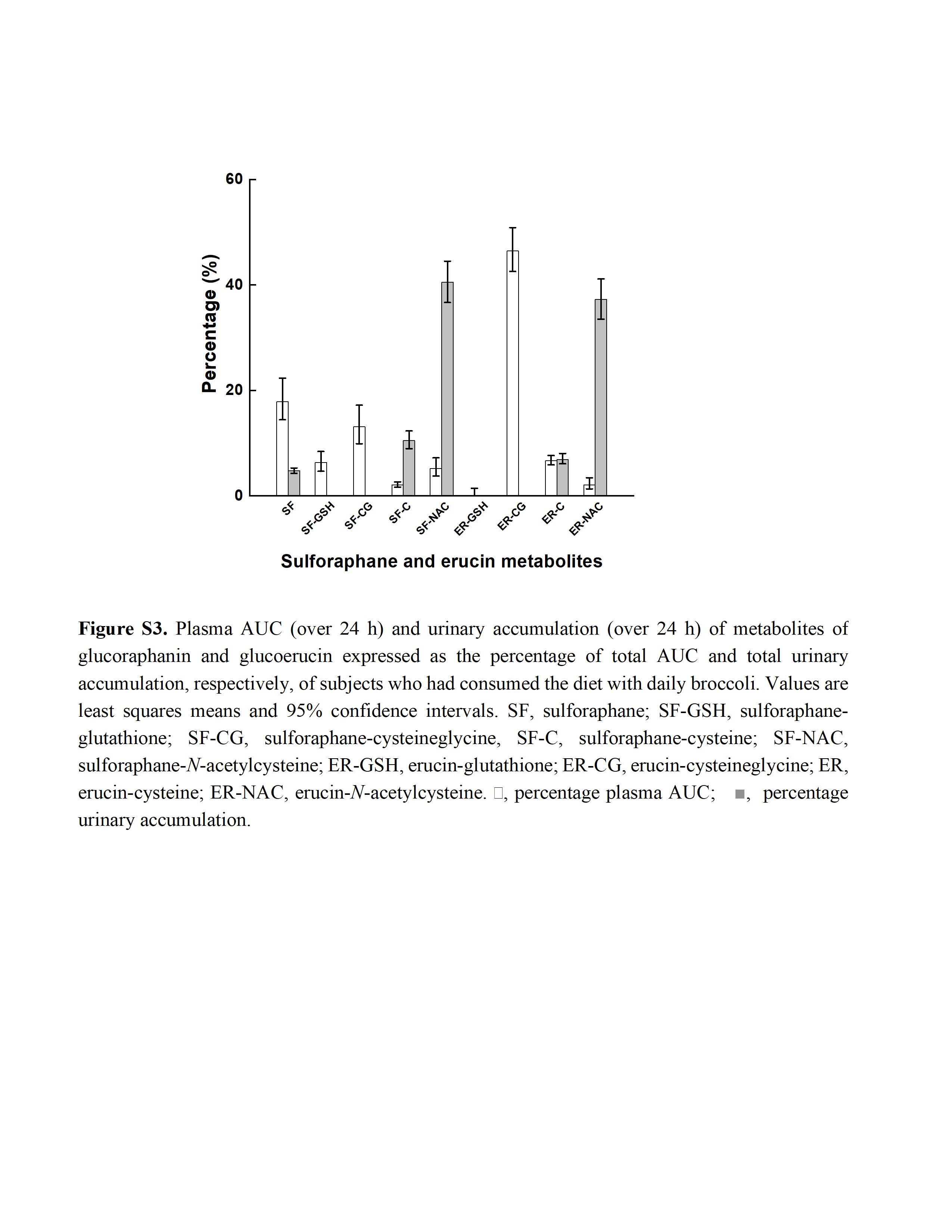

Supplement: Supplementary file 3 [file Image_3.JPEG]
